# A case of nonislet cell tumor hypoglycemia associated with malignant mesothelioma requiring a multifaceted approach for optimal glycemic control

**DOI:** 10.1002/ccr3.5028

**Published:** 2021-10-28

**Authors:** Mariko Ono, Yuki Maeda, Nobuyuki Koyama, Terumasa Nagase, Yoshiya Katsura, Hiroyuki Nakamura, Kazutetsu Aoshiba

**Affiliations:** ^1^ Department of Respiratory Medicine Tokyo Medical University Ibaraki Medical Center Ami Japan; ^2^ Department of Respiratory Medicine Tokyo Medical University Tokyo Japan; ^3^ Department of Clinical Oncology Tokyo Medical University Ibaraki Medical Center Ami Japan; ^4^ Department of Metabolism and Endocrinology Tokyo Medical University Ibaraki Medical Center Ami Japan

**Keywords:** hypoglycemia, insulin‐like growth factor II, mesothelioma

## Abstract

Nonislet tumor hypoglycemia (NICTH) is a rare paraneoplastic syndrome characterized by refractory hypoglycemia, which often requires multifaceted therapy. We reported a case of a patient with pleural malignant mesothelioma and developed NICTH, for which chemotherapy, glucocorticoids, and nutrition were given to achieve optimal glycemic control.

## Graphical abstract

In patients with tumors and hypoglycemia without clear etiology, nonislet cell tumor hypoglycemia (NICTH), which can cause severe refractory hypoglycemia, should be considered. If tumor resection is not feasible, a multifaceted therapeutic approach that includes anticancer drugs, corticosteroids, and nutrition may be required for the management of NICTH.

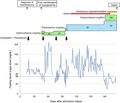

## 1 INTRODUCTION

Nonislet‐cell tumor hypoglycemia (NICTH) is a rare comorbidity that can occur in patients with various tumors, such as solitary fibrosis tumor (SFT), which is the most frequent etiology at a rate of 5% and other more rare tumor etiologies, such as hepatocellular carcinoma, hemangiopericytoma, and fibrosarcoma.^1^, ^2^ However, owing to its rare incidence and lack of awareness, NICTH is often overlooked as a potential cause of hypoglycemia in patients with tumors. Furthermore, NICTH often represents a serious clinical problem, because most patients develop tumor progression, which precludes surgical removal and causes recurrent hypoglycemia. We reported a case of NICTH that developed in a diabetic patient with malignant mesothelioma, and who required chemotherapy, corticosteroids, and nutritional supplementation in order to achieve optimal glycemic control.

## 2 CASE PRESENTATION

A 77‐year‐old Japanese man was admitted to our hospital with a one‐week history of dyspnea on exertion. He had a 10‐year history of well‐controlled diabetes mellitus and had been taking pioglitazone (15 mg/day) and alogliptin (25 mg/day). His occupation was a builder, and he had history of exposure to asbestos 50 years ago and cigarette smoking (0.75 packs per day for 57 years).

**FIGURE 1:**
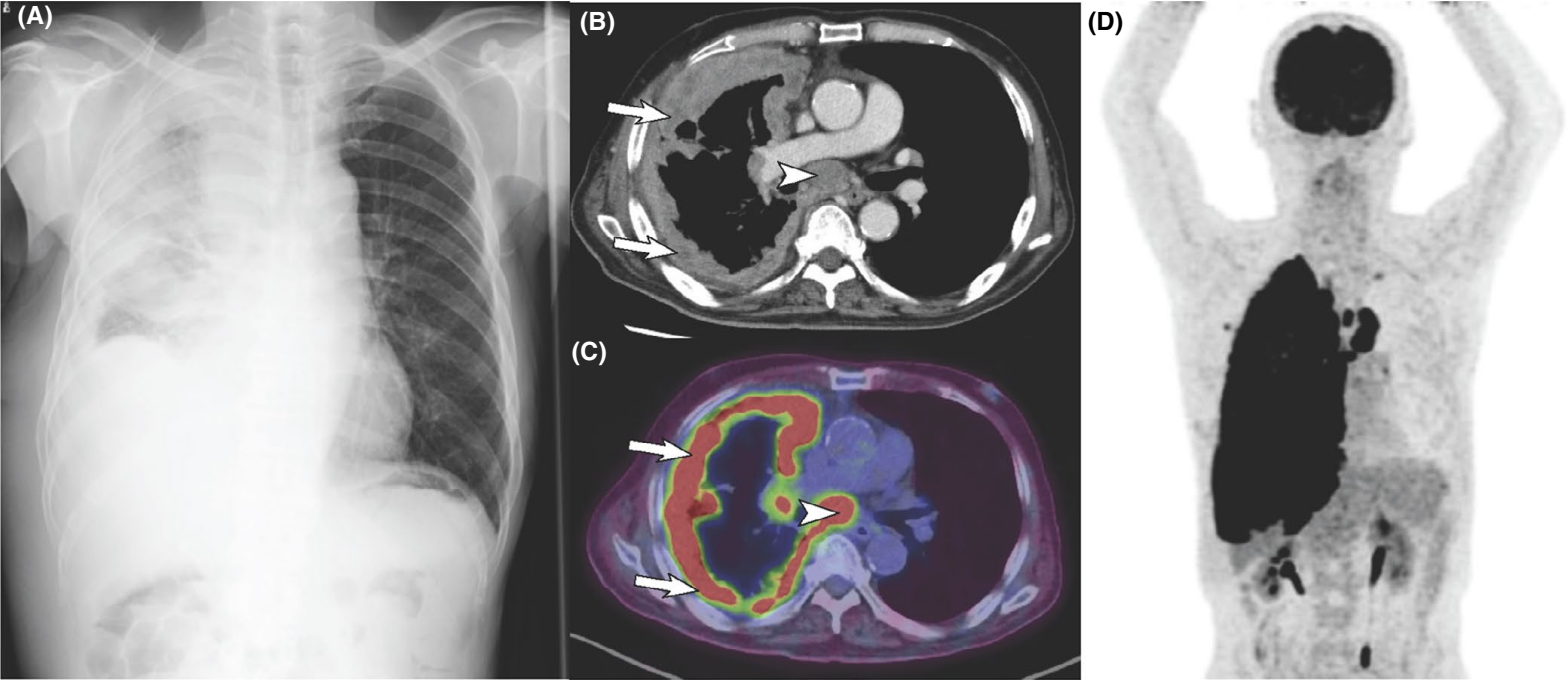
Radiologic findings. (A) Plain chest X‐ray image shows diffuse opacities in the right lung. (B) Contrast‐enhanced chest CT shows diffuse pleural thickening on the right side (*arrows*) and enlargement of mediastinal lymph nodes (*arrowhead*). (C and D) Axial PET‐CT fusion (C) and maximal intensity projection (MIP) images (D) show increased FDG accumulation in the thickened pleura (*arrows*) and enlarged mediastinal lymph nodes (*arrowhead*)

Plain chest X‐ray and computed tomography (CT) of the chest showed diffuse pleural thickening on the right side and enlargement of mediastinal lymph nodes (Figure 1A,B). 18‐Fluoro‐2‐deoxyglucose positron emission tomography and CT (FDG‐PET/CT) revealed increased FDG accumulation in the mediastinal lymph nodes and the right pleura (Figure 1C,D). Pleural fluid cytology showed aggregation of tumor cells, which were proven on cell block immunocytochemistry to be positive for CK 5/6 and calretinin but negative for TTF1, napsin A, p40, and desmin, indicating the diagnosis of malignant mesothelioma. There was no evidence of distant metastasis. The patient was started on a cycle of carboplatin and pemetrexed every 28 days, but the effect of chemotherapy was unremarkable.

At 47 days after admission, the patient experienced hypoglycemia with a blood glucose level of 57 mg/dl, for which he received intravenous glucose. His hypoglycemic event recurred even after withdrawal of hypoglycemic agents. Fasting serum tests showed the following findings: insulin <0.1 μU/ml (normal: 5–15 μU/ml), C‐peptide 1.1 ng/ml (normal: 0.61–2.09 ng/ml), growth hormone (GH) 2.21 ng/ml (normal: ~2.47 ng/ml), insulin‐like growth factor I (IGF I) 30 ng/ml (normal: 48–177 ng/ml), and cortisol 26.4 μg/dl (normal: 7.07–19.6 μg/dl). Serum anti‐insulin antibodies were undetectable.

**FIGURE 2:**
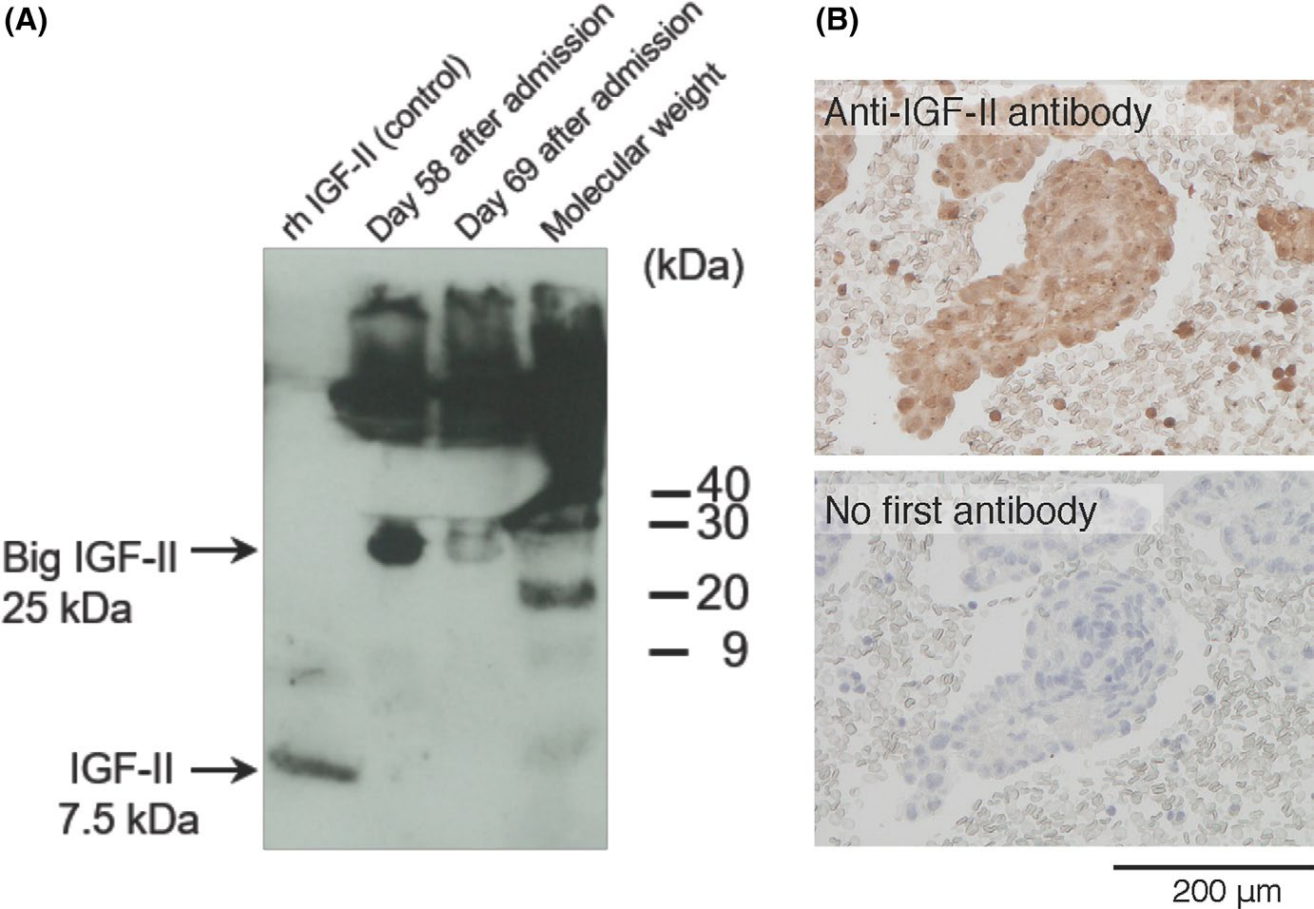
(A) Western blot analysis of the serum samples using anti‐IGF II antibody shows that majority of the IGF II peptide peptides are large (approximately 25 kDa). The molecular weight of recombinant human IGF II (rh IGF II) is 7.5 kDa. (B) Immunocytochemical staining of the cell blocks obtained from the pleural fluid reveals positive immunosignals for the anti‐IGF II antibody in the tumor cells

Based on the findings of recurrent hypoglycemia despite withdrawal of hypoglycemic agents, low serum insulin level, and the presence of malignant tumor, NICTH was suspected. In addition to chemotherapy, oral hydrocortisone therapy (40 mg/day) was initiated on the 48 days after admission. Based on a previous report that NICTH is caused by high‐molecular‐weight insulin‐like growth factor II (big IGF II), which is secreted by tumors,^3^ we performed a Western blot analysis of serum samples using anti‐IGF II antibody. The result revealed that most of the IGF II peptides were big IGF II (approximately 25 kDa compared with the 7.5‐kDa weight of mature IGF II),^4^ allowing us to make a final diagnosis of NICTH (Figure 2A). A positive anti‐IGF II immunostaining of the tumor cells supported the diagnosis of NICTH (Figure 2B).

**FIGURE 3:**
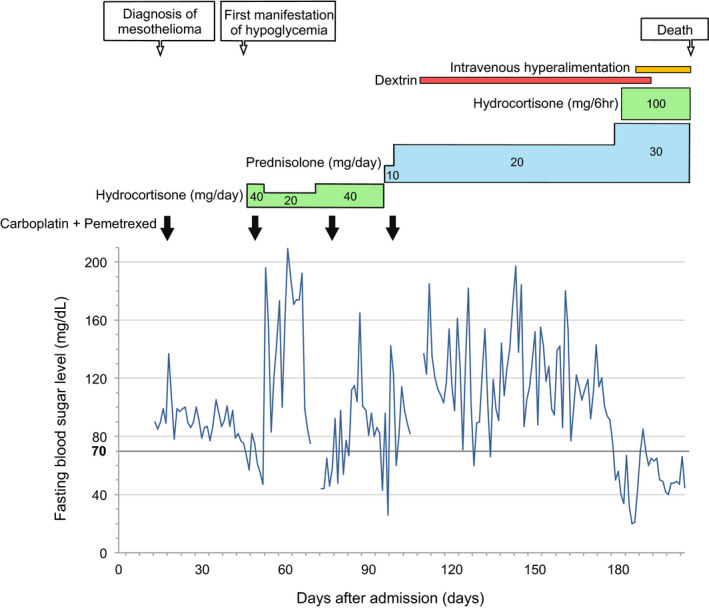
Timeline of the patient's clinical course

After two courses of carboplatin and pemetrexed therapy combined with maintenance hydrocortisone (40 mg/day), the hypoglycemia resolved and the serum level of big IGF II decreased at 69 days after admission (Figure 2A). However, because the tumor grew despite repeated chemotherapy, he needed to receive increasing doses of corticosteroids with up to 30 mg/day of prednisolone/day and 400 mg/day of hydrocortisone in order to control hypoglycemia. In addition, to avert nocturnal hypoglycemia, he received uncooked cornstarch supplement dextrin at 9 g/day at bedtime. Chemotherapy was discontinued after five courses because of poor general condition. Thereafter, the hypoglycemia worsened, and intravenous hyperalimentation was initiated to maintain optimal glycemic control. Although the hypoglycemic symptoms were controlled, the patient died of respiratory failure 157 days after the onset of the hypoglycemic episodes (i.e., 204 days after admission) Figure 3.

## 3 DISCUSSION

IGF II, which is a 7.5‐kDa peptide, is normally produced by the liver and other various tissues. In normal human serum, most of the IGF II peptides bind with the IGF‐binding protein 3 (IGFBP 3) and an acid‐labile subunit (ALS) to form a 150‐kDa ternary complex. Because of its large molecular mass, the ternary complex is unable to pass the capillary membrane.^1^, ^3^, ^4^ In patients with NICTH, however, the tumor secretes incompletely processed precursors of IGF II (i.e., big IGF II: 10–30 kDa), which form a binary complex with IGFBP 3 but do not form a trimer with ALS.^1^, ^3^, ^4^, ^5^ This smaller binary complex has a relatively high capillary permeability and therefore can easily mobilize into tissues and cause hypoglycemia through its action on the insulin receptor or IGF I receptor.^1^, ^3^, ^4^ Moreover, increased IGF II activity suppresses the secretion of insulin, IGF I, and GH; low GH decreases ALS and IGFBP 3 levels, which allow less binding of big IGF II to potentiate hypoglycemia.^1^, ^3^, ^4^ Therefore, as presented in our case, the typical serum laboratory findings in patients with NICTH include low glucose with low levels of insulin, C‐peptide, GH, and IGF 1. In addition to its hypoglycemic action, increased IGF II activity in NICTH may stimulate tumor progression, because IGF II was reportedly a mitogen.^4^

Although NICTH is a rare complication of mesothelioma, up to 8% of all NICTH cases were reportedly accounted for by mesothelioma.^2^ The mainstay treatment of NICTH is complete removal of the tumor.^1^, ^3^ However, most patients with mesothelioma are inoperable at diagnosis and need a multidisciplinary approach that aims to controlling hypoglycemia, because the available chemotherapy regimens achieve poor results. Corticosteroids have been shown to be the most effective for NICTH by stimulating gluconeogenesis, suppressing the production of big IGF II, and correcting the biochemical abnormalities that involve the GH–IGF axis.^3^, ^6^ In our patient, the first course of chemotherapy with carboplatin and pemetrexed failed to prevent the onset of hypoglycemic episodes. However, the second course of the same regimen in combination with daily supplementation of hydrocortisone (40 mg/day) successfully prevented the development of hypoglycemia and reduced the serum level of big IGF II, suggesting the beneficial effect of corticosteroids on NICTH. In this patient, despite the subsequent progressive tumor growth, hypoglycemia was fairly well‐controlled by increasing the doses of corticosteroids. Although the tumor size was not significantly reduced, chemotherapy with carboplatin and pemetrexed also played a beneficial role in ameliorating hypoglycemia. In fact, hypoglycemia occurred more often immediately before than after the course of chemotherapy and worsened further after than before discontinuation of chemotherapy.

Adequate caloric support is also important for the management of NICTH. In addition to oral glucose and/or intravenous glucose‐containing fluids as necessary, our patient ingested uncooked cornstarch (i.e., dextrin) at bedtime to prevent nocturnal hypoglycemia through the slow release of glucose. When oral food intake was restricted because of the deteriorated general condition, he eventually received intravenous hyperalimentation with high glucose concentration to prevent hypoglycemia. This comprehensive approach to optimize glycemic control avoided the occurrence of severe hypoglycemic episodes and helped keep the patient conscious until he died of respiratory failure. The treatment modalities that were utilized in the case reports on NICTH but were not tried in our patient include recombinant human GH, glucagon, and octreotide.^1^, ^4^

In patients with tumors and hypoglycemia without clear etiology, NICTH should be considered. Although a rare complication of mesothelioma, NICTH could cause severe refractory hypoglycemia, because complete tumor resection is not feasible in most cases of mesothelioma. In the management of such NICTH cases, a multifaceted therapeutic approach, including anticancer drugs, corticosteroids, and nutrition, uncooked cornstarch, and hyperalimentation may be required.

## CONFLICTS OF INTEREST

The authors have no conflicts of interest to declare.

## AUTHOR CONTRIBUTIONS

MO and KA: wrote the first draft. YM, TN, YK, NK, and HN: contributed to the clinical management of the patient and revised the manuscript.

## ETHICAL STATEMENT

This case report meets the standards of Tokyo Medical University ethical committee. All personal identifiers were removed from the manuscript.

## CONSENT

Informed consent from the patient was obtained for publication of the case report.

## Data Availability

The data that support the findings of this study are available on request from the corresponding author. The data are not publicly available due to privacy or ethical restrictions.

## References

[1] Bodnar TW , Acevedo MJ , Pietropaolo M . Management of non‐islet‐cell tumor hypoglycemia: a clinical Review. J Clin Endocrinol Metab. 2014;99(3):713‐722. 24423303 10.1210/jc.2013-3382 PMC5393479

[2] Dynkevich Y , Rother KI , Whitford I , et al. Tumors, IGF‐2, and hypoglycemia: insights from the clinic, the laboratory, and the historical archive. Endocr Rev. 2013;34(6):798‐826. 23671155 10.1210/er.2012-1033

[3] de Groot JWB , Rikhof B , van Doorn J , et al. Non‐islet cell tumour‐induced hypoglycaemia: a review of the literature including two new cases. Endocr Relat Cancer. 2007;14(4):979‐993. 18045950 10.1677/ERC-07-0161

[4] van Doorn J . Insulin‐like growth factor‐II and bioactive proteins containing a part of the E‐domain of pro‐insulin‐like growth factor‐II. Bio Factors. 2020;46(4):563‐578. 10.1002/biof.1623 PMC7497164 32026557

[5] Daughaday WH , Emanuele MA , Brooks MH , et al. Synthesis and secretion of insulin‐like growth factor II by a leiomyosarcoma with associated hypoglycemia. N Engl J Med. 1988;319(22):1434‐1440. 3185662 10.1056/NEJM198812013192202

[6] Teale JD , Marks V . Glucocorticoid therapy suppresses abnormal secretion of big IGF‐II by non‐islet cell tumors inducing hypoglycemia (NICTH). Clin Endocrinol (Oxf). 1998;49(4):491‐498. 9876347 10.1046/j.1365-2265.1998.00564.x

